# Polyacrylamide Microspheres-Derived Fe_3_C@N-doped Carbon Nanospheres as Efficient Catalyst for Oxygen Reduction Reaction

**DOI:** 10.3390/polym11050767

**Published:** 2019-05-01

**Authors:** Ming Chen, Yu Jiang, Ping Mei, Yan Zhang, Xianfeng Zheng, Wei Xiao, Qinliang You, Xuemin Yan, Haolin Tang

**Affiliations:** 1 College of Chemistry and Environmental Engineering, Yangtze University, Jingzhou 434023, Hubei, China; Mingchen126@163.com (M.C.); librajiangyu@163.com (Y.J.); PingMei@126.com (P.M.); YanZhang@126.com (Y.Z.); Xianfzheng@163.com (X.Z.); WeiXiao@126.com (W.X.); 2 Key Laboratory of Optoelectronic Chemical Materials and Devices, Ministry of Education, School of Chemical and Environmental Engineering, Jianghan University, Wuhan 430056, China; QinliangYou@126.com; 3 State Key Laboratory of Advanced Technology for Materials Synthesis and Processing, Wuhan University of Technology, Wuhan 430070, Hubei, China

**Keywords:** polyacrylamide microspheres, N-doped carbon, Fe_3_C, core-shell structure, oxygen reduction reaction

## Abstract

High-performance non-precious metal catalysts exhibit high electrocatalytic activity for the oxygen-reduction reaction (ORR), which is indispensable for facilitating the development of multifarious renewable energy systems. In this work; N-doped carbon-encapsulated Fe_3_C nanosphere ORR catalysts were prepared through simple carbonization of iron precursors loaded with polyacrylamide microspheres. The effect of iron precursors loading on the electrocatalytic activity for ORR was investigated in detail. The electrochemical measurements revealed that the N-doped carbon-encapsulated Fe_3_C nanospheres exhibited outstanding electrocatalytic activity for ORR in alkaline solutions. The optimized catalyst possessed more positive onset potential (0.94 V vs. reversible hydrogen electrode (RHE)), higher diffusion limiting current (5.78 mA cm^−2^), better selectivity (the transferred electron number *n* > 3.98 at 0.19 V vs. RHE) and higher durability towards ORR than a commercial Pt/C catalyst. The efficient electrocatalytic performance towards ORR can be attributed to the synergistic effect between N-doped carbon and Fe_3_C as catalytic active sites; and the excellent stability results from the core-shell structure of the catalysts.

## 1. Introduction

With the limited supply of fossil fuels and growing concern about environmental problems, the development of renewable energy and efficient energy conversion systems has become more important than ever. Fuel cells are regarded as one of the most promising energy conversion devices accessible nowadays [1,2,3,4,5,6]. Among various fuel cells, proton exchange membrane fuel cells (PEMFCs) and polymer electrolyte fuel cells (PEFCs) are considered as advanced electrochemical energy conversion systems due to their advantages of fast electrode reaction kinetics, high conversion efficiency and environmental friendliness [7,8]. In fuel cells, small molecule fuels are oxidized at the anode and concurrently oxygen is reduced at the cathode [9]. Efficient catalysts are necessary due to the sluggish kinetics of the oxygen-reduction reaction (ORR) at the cathode which is of great limit of the energy conversion efficiency of fuel cells. Pt and Pt-based materials have been used extensively as effective catalysts for ORR, which have extremely low overpotential as well as high current density in the ORR process [9,10,11,12]. However, the high price as well as the relatively low stability has been the main bottlenecks that hinder their broader commercialization [13,14]. Therefore, the development of low-cost and high-performance alternatives, especially non-precious metal catalysts, have aroused extensive research interest. Among these potential alternatives, N-doped carbon and transition metal species have attracted much attention due to the relatively low cost and promising electrocatalytic activity to ORR [15,16,17,18,19]. Furthermore, specific surface area and structure of the catalysts were found to determine the accessibility of the active sites and can largely improve the ultimate catalytic performance [20,21,22,23,24]. Recently, non-precious metal catalysts based on metal carbides, such as Fe_3_C, coated with N-doped carbon nanostructures, have been proved to be very efficient for ORR. The N-doped carbon-encapsulated structures offer a specific synergistic effect of Fe_3_C active sites and N-doped carbon matrix [25,26,27,28]. In addition, this kind of structure partly prevents metal composite from corrosion in electrolytes, which is necessary for long-term performance [29]. Typically, the N-doped carbon encapsulated Fe_3_C composite was prepared by Fe-MOF or a molecular precursor containing Fe, nitrogen and carbon precursor [30,31,32]. G. Ren [33] and R. Zhong [34] reported that porous core-shell Fe_3_C embedded N-doped carbon nanofibers were synthesized by the electrospinning method. The resulting nanofibers catalysts showed excellent ORR activities and high stabilities, which were attributed to well-dispersed nanocrystalline Fe_3_C active sites, high N-doping level, large surface areas, and a one-dimensional carbon nanostructure. Therefore, the development of a facile strategy for designing and synthesizing core-shell structured catalysts with high surface areas and abundant active sites is highly desirable for the further development of the N-doped carbon encapsulated metal composite ORR catalysts.

Herein, we report a facile and controllable fabrication of N-doped microporous carbon encapsulated Fe_3_C nanospheres derived from iron precursor-loaded polyacrylamide microspheres. Polyacrylamide microspheres act as the carbon and nitrogen precursor and possess excellent hydroscopicity, which can adsorb iron salts and ensure their uniform distribution. The amounts of the active sites are regulated by adsorbing different amount of iron precursors before carbonization of polyacrylamide microspheres and the porous structures are obtained by activation of KOH. The obtained catalysts are highly microporous with well-dispersed Fe_3_C nanospheres in the N-doped graphite carbon matrix. As expected, Fe_3_C@N-doped microporous carbon nanospheres exhibited better ORR electrocatalytic activity in alkaline media compared to the commercial Pt/C catalyst.

## 2. Experimental Section

### 2.1. Materials and Reagents

Tween-60 (C_32_H_62_O_10_), N,N′-Methylenebisacrylamide (C_7_H_10_N_2_O_2_, 98.0%), ammonium persulfate ((NH4)_2_S_2_O_8_, 98.5%), acrylamide (C_3_H_5_NO, 98.0%), Potassium hydroxide (KOH, >96.0%) and isopropanol (C_3_H_8_O, >99.7%) were obtained from Sinopharm Chemical Reagent Co., Ltd. (Shanghai, China). Ferroporphyrin (C_34_H_32_ClFeN_4_O_4_, 98.0%), Span-80 (C_24_H_44_O_6_) and mineral oil were purchased from Shanghai Yuanye Biological Co., Ltd, Shanghai, China). 20 wt % Pt/C catalyst and 5 wt % Nafion solution were obtained from Shanghai Macklin Biochemical Co., Ltd, Shanghai, China). All the chemicals were used as received without further purification.

### 2.2. Synthesis of Fe_3_C@N-Doped Carbon Nanospheres

Polyacrylamide microspheres were firstly synthesized by inverse emulsion polymerization. Briefly, 50 mL mineral oil and 6.2 g of Span-80 were mixed for 10 min to act as oil phase. 20.0 g of acrylamide monomer, 1.8 g of Tween-60 and 1 mL N,N′-Methylenebisacrylamide solution (0.04 wt %) were dissolved into 25 mL deionized water and then 1 mL ammonium persulfate solution (0.4 wt %) was dripped after stirring for 5 minutes, the mixture solution was served as water phase. The water phase was added dropwise into the oil phase with stirring at the speed of 600 r/min. After 30 min of vigorous stirring, the mixed solution was put into a 55 °C water bath and the N_2_ was aerated continuously to remove the air for about 40 min. Then the mixture was kept in a 55 °C water bath with stirring at the speed of 300 r/min in nitrogen atmosphere for 3 h to obtain crosslink-polyacrylamide reverse-phase microemulsion. The resulting w/o microemulsion was poured into excess acetone for demulsification. After washing with deionized water and absolute ethyl alcohol, the collected solids were dried at 70 °C overnight to obtain polyacrylamide microspheres. In order to obtain carbonization yield of polyacrylamide microspheres, in preliminary experiment, 2 g of dried polyacrylamide microspheres was pyrolyzed under flowing N_2_ for 1 h at 900 °C with a heating rate of 5 °C min^−1^ and 0.4 g of carbon was collected. This indicates that the carbonization yield of polyacrylamide microspheres was about 20%.

Fe_3_C@N-doped carbon nanospheres were prepared by direct carbonization of iron precursors-loaded polyacrylamide microspheres. In a typical preparation process, a certain amount of ferroporphyrin and 1.6 g of KOH solid were dissolved in 15 mL deionized water, the mixture solution was completely absorbed by 4 g dried polyacrylamide microspheres. The KOH was added to dissolve the Ferroporphyrin and then activate the carbon. The sample was dried at 70 °C for 24 h and calcinated at 900 °C for 1 h filled with N_2_ atmosphere and the ramping rate was 5 °C min^−1^. The resulting dark powder was washed with deionized water for several times to remove alkali metal K which was produced during the activation and calcination process, followed by drying at 70 °C overnight. The final as-synthesized product has been obtained and labeled as Fe_3_C@N/C-x, and x represents the mass ratio of ferroporphyrin and carbon according to the theoretical dosage. For comparison, a series of Fe_3_C@N/C-x composites were also prepared via the same procedure except for the difference of ferroporphyrin additive amounts. These as-synthesized catalysts with various ferroporphyrin additive amounts were labeled as Fe_3_C@N/C-0, Fe_3_C@N/C-0.5, Fe_3_C@N/C-1, Fe_3_C@N/C-2, respectively.

### 2.3. Characterizations

Transmission electron microscope (TEM) images were collected using a JEM-2100F TEM (Tokyo, Japan), XRD measurements were measured with a D8 Advance X-ray diffractometer from Bruker AXS Company (Karlsruhe, Germany). X-ray photoelectron spectroscopy (XPS) analysis was recorded on a VG Multilab 2000 X-ray photoelectron spectrometer (VG Scientific, Waltham, MA, USA). The pore structure analysis was performed through nitrogen adsorption and desorption isotherm measurements (BET, ASAP 2020, Micromeritics). The dynamic function theory (DFT) method was applied to calculate the distribution of the micropore size.

### 2.4. Electrochemical Measurements

To evaluate ORR activity, the electrochemical performance of Fe_3_C@N-doped microporous carbon nanospheres catalysts were performed in a standard three-electrode glass cell with N_2_ or O_2_-saturated 0.1M KOH solution at room temperature on the electrochemical workstation (CHI660E, CHI instrument) using a glassy carbon electrode (GCE, of 5.00 mm in diameter) as working electrode, a platinum wire as counter electrode, and Hg/HgO electrode as reference electrode, respectively. The electrochemical properties of as-synthesized catalysts were referred to the Hg/HgO electrode. The Hg/HgO reference electrodes were calibrated with respect to the reversible hydrogen electrode (RHE) before measurement [2]. The calibration values were *E*(RHE) = *E*(Hg/HgO) + 0.89 V in 0.1M KOH. 5.0 mg of as-synthesized catalysts power, 0.1 mL of deionized water, 0.9 mL of isopropanol and 0.02 mL of 5 wt % nafion solution was ultrasonically mixed to form the ink of the catalysts. The GCE was modified with 20 µL of the ink to serve as the working electrode. Cyclic voltammetry (CV) and linear sweep voltammetry (LSV) were carried out to evaluate the ORR performances. CV curves were obtained in N_2_ or O_2_-saturated 0.1M KOH electrolyte solutions without any rotation. The CV measurement data were carried out in the potential range from −0.6 V to 0.4 V at a sweep rate of 50 mV s^−1^, and LSV measurements were carried out at a scan rate of 5 mV s^−1^ in the potential range from -0.8 V to 0.2 V under various electrode rotation rates (400, 800, 1200, 1600, 2000 rpm) in O_2_-saturated 0.1M KOH electrolyte solutions. All samples were also tested in N_2_-saturated for comparison. Chronoamperometric measurements for each as-synthesized catalyst were investigated in O_2_-saturated 0.1M KOH (−0.3 V vs. Hg/HgO) at room temperature. The ORR process kinetics was analyzed using the Koutecky–Levich (K–L) equation.

## 3. Results and Discussion

**Figure 1:**
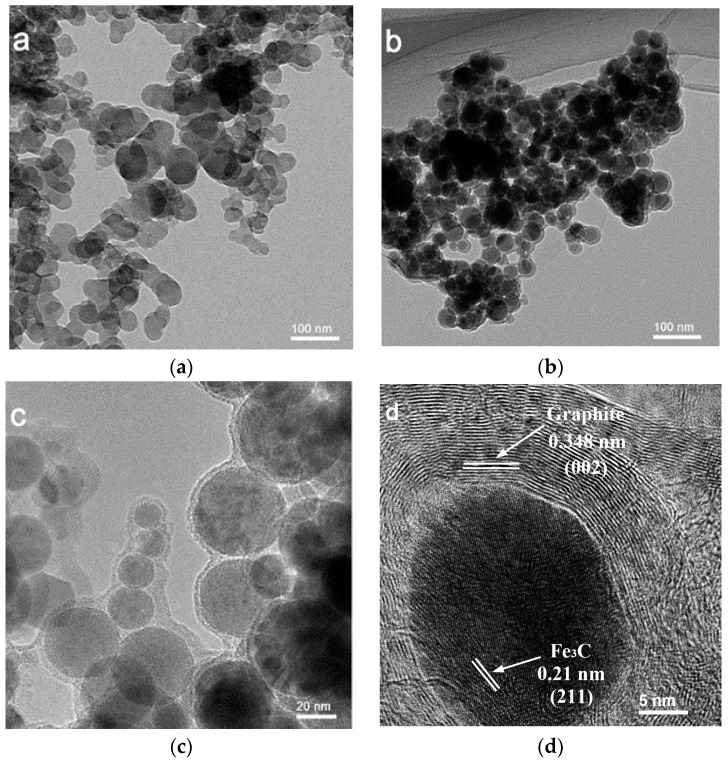
(**a**) Transmission electron microscope (TEM) image of polyacrylamide microspheres; (**b**,**c**) TEM and (**d**) high-resolution TEM (HRTEM) image of Fe_3_C@N/C-1.

The morphology of polyacrylamide microspheres and the N-doped carbon encapsulated Fe_3_C nanospheres catalysts were characterized by TEM. Figure 1a shows the surface morphology of polyacrylamide microspheres. The results showed that the polyacrylamide microspheres are aggregates of spherical particles with an average diameter of about 30 nm. After adsorbing iron precursors and carbonization, the spherical morphology of samples can be maintained. The TEM images of Fe_3_C@N/C-1 were shown in Figure 1b,c. It can be observed that the core-shell structured catalysts were successfully synthesized. The high-resolution TEM (HRTEM) results in Figure 1d showed that the spacing between adjacent lattice fringe in nanospheres core was 0.21 nm, corresponding to the (211) crystal planes of Fe_3_C. The lattice fringe in outer shell was 0.348 nm, corresponding to the graphite (002) plane. It indicated that the Fe_3_C nanospheres were obtained and wrapped within graphite carbon layer during the carbonization process.

**Figure 2:**
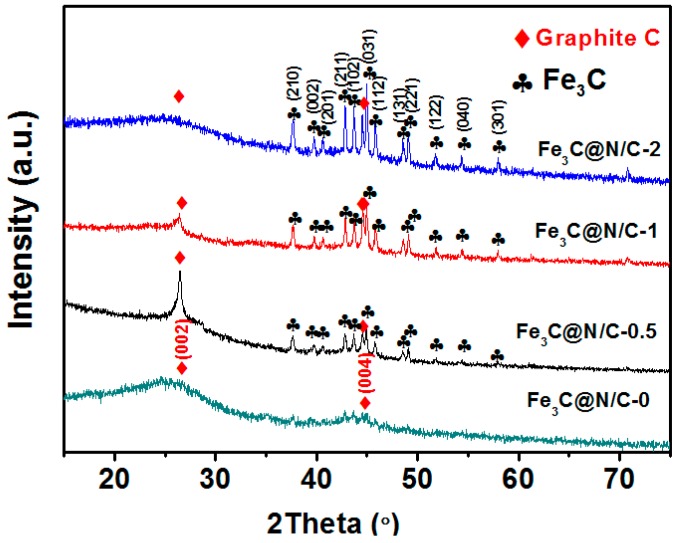
X-ray diffraction (XRD) patterns of Fe_3_C@N/C-x samples.

In order to further identify the crystal structure of samples, X-ray diffraction (XRD) patterns of these samples were recorded and shown in Figure 2. The major peaks located at 37.8°, 43.1°, 43.1°, 43.9°, 44.9°, 46.0°, 48.7° and 49.3° correspond to the Fe_3_C phase [35]. In addition, the peaks at 26.4° and 44.0° were assigned to the (002) and (004) reflection of graphitic carbon [26]. There are only broad graphitic carbon diffraction peaks in Fe_3_C@N/C-0 sample. With the introduction of ferroporphyrin, the graphitic carbon and Fe_3_C crystalline phases coexisted in the other samples. The relative intensity of Fe_3_C diffraction peaks increased with the iron content further enhanced in the synthesis process, indicating an increased ratio of Fe_3_C. The Fe_3_C nanocrystalline should originate from the reduction of iron precursor by carbon during the pyrolysis process. In the synthesis process of catalysts, ferroporphyrin was firstly dissolved in the KOH solution and homogeneously adsorbed in the polyacrylamide microspheres. Iron compound could be reduced by carbon to form metallic iron at high temperature, and these metallic iron atoms contribute to the catalytic graphitization of carbon [36]. When the iron atoms concentration is high enough, it would aggregate to form crystalline irons which react with the carbon atom to form a graphite layer-encapsulated Fe_3_C crystal during the process of pyrolysis and cooling. With the growth of Fe_3_C crystal, it is difficult for the large-size Fe_3_C crystal to make the inside carbon atoms diffuse onto the surface. Therefore, the relative intensity of graphitic carbon diffraction peaks weakens with the increase of iron content in the catalysts [35,36,37,38,39].

**Figure 3:**
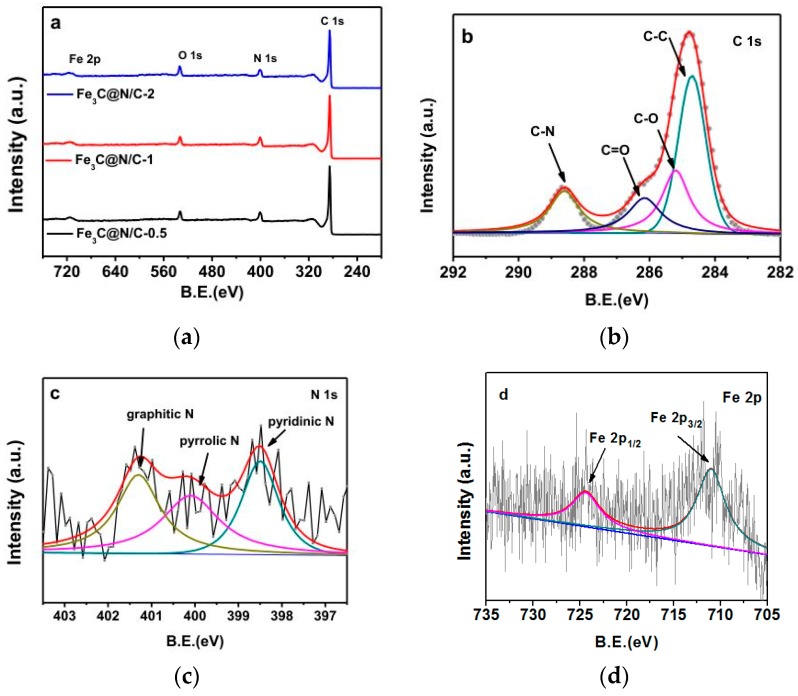
(**a**) X-ray photoelectron spectroscopy (XPS) survey spectra of Fe_3_C@N/C-x samples. (**b**) C 1s, (**c**) N 1s and (**d**) Fe 2p spectra of Fe_3_C@N/C-1.

XPS was conducted to illustrate the surface elements of catalysts. XPS spectra of Fe_3_C@N/C catalysts are exhibited in Figure 3a, which revealed the existence of C, O, Fe and N in catalysts. There is only a weak Fe peak was detected in catalysts, which could be resulted from the coverage of graphitic layers on the Fe_3_C surface [33,39]. The high-resolution C 1s spectrum of Fe_3_C@N/C-1 shown in Figure 3b can be deconvoluted into four individual peaks that are assigned to C–C (284.6 eV), C–O (285.2 eV), C=O (286.5 eV), C–N (288.2 eV), respectively. The N 1s spectrum of Fe_3_C@N/C-1 shown in Figure 3c can be divided into three peaks, assigned to pyridinic N (398.4 eV), pyrrolic N (400.1 eV), graphitic N (401.2 eV), suggesting that nitrogen has been doped into the carbon authentically. It is generally believed that pyridinic N and graphitic N can serve as the efficient active sites for ORR. Pyridinic N increases the spin density and the density of π states of the carbon atoms near the Fermi level which could enhance the reduction of O_2_, while graphitic N can increase the conductivity of the catalysts [35,40,41]. In the case of the high-resolution Fe 2p spectrum (Figure 3d), the peaks at 711.0 eV and 724.4 eV are consistent with Fe 2p_3/2_ and Fe 2p_1/2_ states, respectively [42,43,44]. Fe, C and N content, N/C weight ratios and the relative atomic amount of N species in all of Fe_3_C@N/C-x samples are shown in Table 1. The amount of Fe incorporated into the final catalyst increases with the enhancement of ferroporphyrin loading during the synthesis process, while the amount of N declines slightly at the same time. In all cases, graphitic N and pyridinic N are dominant species. In general, with the increase of the amount of Fe_3_C catalytic active sites, the amount of N-doped carbon catalytic active sites declines. 

**Figure 4:**
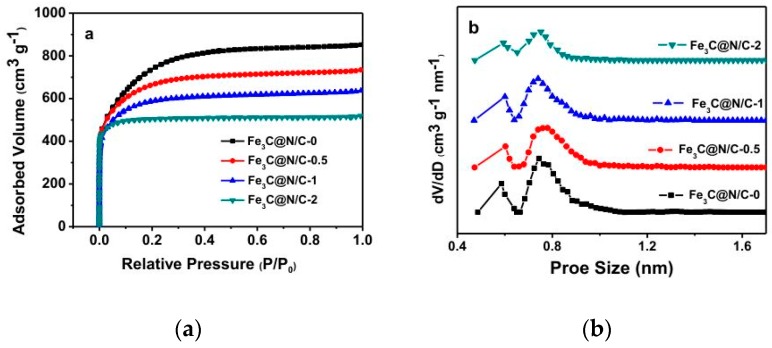
(**a**) Nitrogen adsorption and desorption curves. (**b**) pore size distribution of Fe_3_C@N/C-x samples.

The nitrogen adsorption and desorption isotherms were conducted to investigate the pore structure of these catalyst samples and the results are shown in Figure 4a. The nitrogen adsorption and desorption isotherm curves of all catalysts reveal a Type-I sorption isotherm with no hysteresis, manifesting the microporous structure of the Fe_3_C@N/C-x samples. In the synthesis process, KOH was used not only as an alkaline medium to dissolve ferroporphyrin, but also as an activator to form micropores. The abundant micropores and high specific surface area can improve the accessibility of electrolyte and oxygen to the active sites. The specific surface area and pore volume of all samples are shown in Table 2. With the increase of iron content, the total surface area of Fe_3_C@N/C-x catalysts reduce from 2484 to 1687 m^2^ g^−1^. Figure 4b illustrates the pore size distribution (PSD) curves of Fe_3_C@N/C-x catalysts. The results of PSDs are greatly distributed in the microporous region which is less than 2.0 nm and the peaks are mostly 0.78 nm and 0.59 nm.

**Figure 5:**
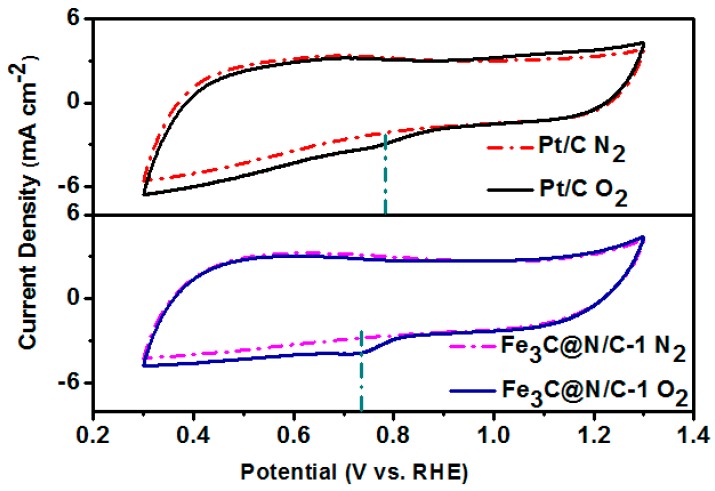
Cyclic voltammetry (CV) curves of Fe_3_C@N/C-1 catalyst and 20 wt % Pt/C in N_2_ and O_2_-saturated in 0.1 M KOH.

**Figure 6:**
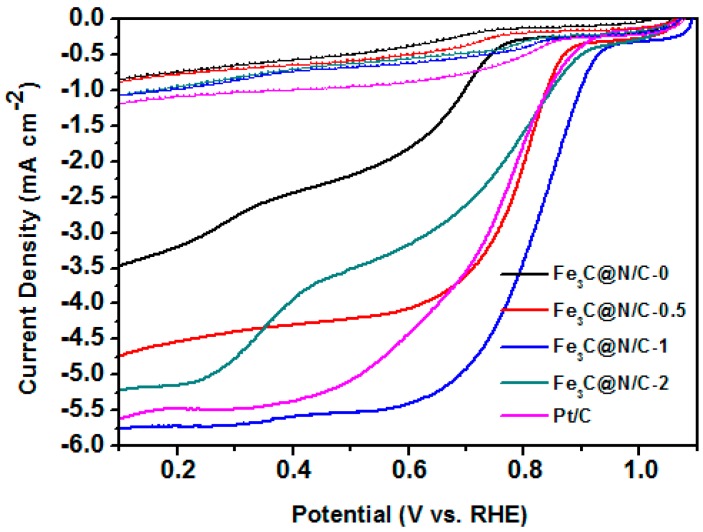
Linear sweep voltammetry (LSV) curves of Fe_3_C@N/C-x samples and Pt/C catalyst at the rotation speed of 1600 rpm in O_2_ (solid lines) and N_2_-saturated (dotted lines).

CV and LSV measurements were performed to study the electrochemical activity of these catalysts. Figure 5 shows the CV curves of Fe_3_C@N/C-1 catalyst and 20 wt % Pt/C in N_2_ and O_2_-saturated 0.1 M KOH solution. Well-defined oxygen reduction peak for Fe_3_C@N/C-1 catalyst and 20 wt % Pt/C could be seen in the CV curves when saturating the alkaline solution with O_2_, illustrating the pronounced ORR activity of Fe_3_C@N/C-1 catalyst. LSV curves were carried out on a rotating disk electrode (RDE) for the further evaluation of the electrocatalytic activity of catalyst samples for ORR. The LSV measurement was tested in O_2_-saturated 0.1M KOH solution at a rotation rate of 1600 rpm with a potential scan rate of 5 mV s^−1^ (the solid line). All samples were also tested in N_2_-saturated for comparison (the same colour scheme as the dotted line). As shown in Figure 6, the Fe_3_C@N/C-1 catalyst with onset potential of 0.94 V (vs. RHE) and diffusion-limiting current of 5.78 mA cm^−2^ reveals the remarkable catalytic performance towards ORR compared to the benchmark Pt/C (0.91 V and 5.55 mA cm^−2^ correspondingly). By contrast, the Fe_3_C@N/C-0 catalyst shows the lowest catalytic activity to ORR. It is reported that the encapsulated metal compound’s nanoparticles could produce host–guest electronic interaction and change the local work function of the carbon shell, creating additional active sites to ORR [33]. Doping of nitrogen into the carbon shell could further improve the catalytic activity of these core-shell structured catalysts by modifying the electronic properties of carbon surface. In addition, there is a general agreement that N-doped graphitic carbon is playing an important role in enhancing the conductivity of the catalysts which is essential for ORR. Therefore, the enhanced electrocatalytic performance of Fe_3_C@N/C-1 can be attributed to the synergistic effect between N-doped carbon and Fe_3_C as catalytic active sites. It should be noted that Fe_3_C@N/C-2 showed obviously lower electrocatalytic activity than that of Fe_3_C@N/C-1, due to the reduced specific surface area and depressed graphitization degree of the carbon shell in the Fe_3_C@N/C-2 catalyst. In Table 3 below, the performance of the Fe_3_C@N/C-1 catalyst is compared to some other Fe_3_C-based electrocatalysts reported in literature. Overall, the Fe_3_C@N/C-1 catalyst possessed comparative onset potential with other Fe_3_C based electrocatalysts. Moreover, the diffusion limiting current of the Fe_3_C@N/C-1 catalyst was higher than that of most catalysts, indicating the excellent electrochemical activity towards ORR.

**Figure 7:**
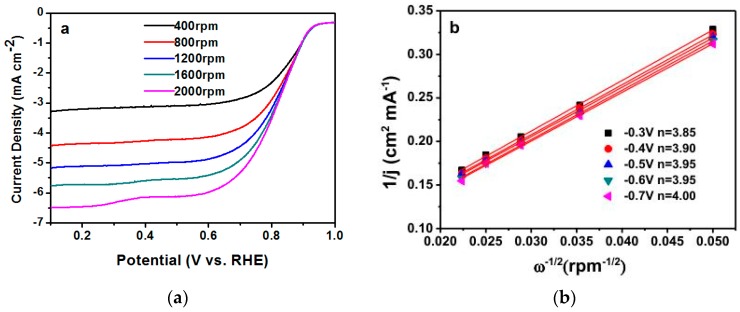
(**a**) LSV curves of Fe_3_C@N/C-1 catalyst with different rotating speed from 400 to 2000 rpm. (**b**) Koutecky–Levich (K–L) plots of Fe_3_C@N/C-1 catalyst calculated from Figure 7a.

**Figure 8:**
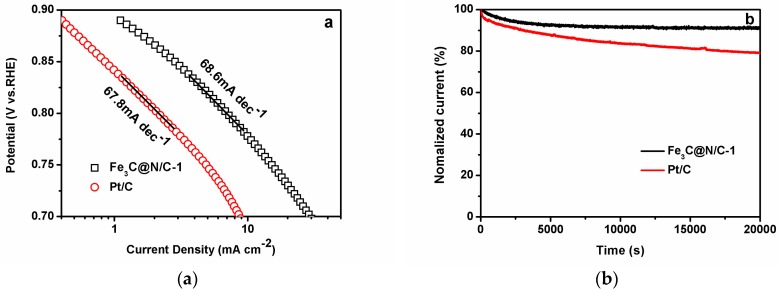
(**a**) Tafel plots of Fe_3_C@N/C-1 catalyst and Pt/C obtained from the rotating disk electrode (RDE) measurements. (**b**) current-time chronoamperometric response of Fe_3_C@N/C-1 catalyst and Pt/C in O_2_-saturated 0.1 M KOH at −0.3 V for 20000s.

RDE measurement of Fe_3_C@N/C-1 catalyst was evaluated under different rotation speeds from 400 rpm to 2000 rpm and the results are shown in Figure 7a. The Koutecky–Levich plots (Figure 7b) were calculated by the recorded reaction currents at −0.3 V on the LSV curves of the Fe_3_C@N/C-1 catalyst under various rotating speeds to study the reaction kinetics of this catalyst. The electron-transfer numbers of the Fe_3_C@N/C-1 catalyst were calculated by the K–L equation [47]. The n value for Fe_3_C@N/C-1 catalyst count from the slope of K–L plots is 3.85–4.00, which is close to 4, suggesting the Fe_3_C@N/C-1 catalyze ORR process through a quasi-four-electron process. H_2_O_2_ was released during 2e^−^ process which degrades the membrane electrolyte; therefore, the 4e^−^ process is desired for a fuel cell [48,49,50,51]. The calculated Tafel slope is shown in Figure 8a, exhibiting a similar Tafel slope for the Fe_3_C@N/C-1catalyst (68.62 mV per decade) and Pt/C (67.8 mV per decade), indicating the similar reaction kinetics of ORR on the Fe_3_C@N/C-1 catalyst surface and the first electron is probably the rate-determining step.

The stability of catalyst is necessary for the practical application of fuel cells. The chronoamperometric measurements were investigated to evaluate the durability of the Fe_3_C@N/C-1 catalyst. The glassy-carbon electrode modified with Fe_3_C@N/C-1 and commercial Pt/C were tested at constant voltage of −0.3 V in an O_2_-saturated 0.1M KOH aqueous solution with rotation rate of 1600 rpm for 20,000 s. The current-time response is shown in Figure 8b. As we can see, the current-time chronoamperometric response for commercial Pt/C exhibited a rapid current decrease and the current loss is about 25% after 20,000 s. In comparison, the current retention for the Fe_3_C@N/C-1 catalyst is 94% after 20,000 s, implying the excellent stability under working conditions. The excellent stability of Fe_3_C@N/C-1 catalyst may in virtue of the appearance of the sufficient outer graphene layer. Thus, it can be concluded that the Fe_3_C@N-doped microporous carbon nanosphere catalyst is a promising alternative for costly Pt-based electrocatalysts to apply in fuel cells.

## 4. Conclusions

In summary, Fe_3_C@N-doped microporous carbon nanosphere catalysts were facilely and successfully synthesized by using polyacrylamide microspheres as a carbon and nitrogen resource and ferroporphyrin as an iron resource. The composite and nanostructure of catalysts were characterized by TEM, XRD, XPS and BET analysis and the electrochemical activity of these catalysts were investigated by CV and LSV measurements. The Fe_3_C@N/C-1 catalyst showed excellent ORR activity and long-term stability in alkaline medium, with an onset potential and diffusion limiting current of 0.94 V vs. RHE and 5.78 mA cm^−2^, which is superior to that of commercial Pt/C catalyst. The outstanding performance is attributed to the synergistic effect between N-doped carbon and Fe_3_C as catalytic active sites, and the abundant microporous structure can improve the accessibility of electrolyte and oxygen to active sites. In addition, the carbon shell prevents Fe_3_C active sites from leaching out. These results show that this kind of core-shell catalyst could be a promising alternative to Pt-based catalysts for the further development of durable and efficient ORR catalysts.

## Figures and Tables

**Table 1:** Fe, C and N content, N/C weight ratios and the relative atomic amount of N species in all of the Figure 3 samples derived from XPS analyses.

Sample	Weight Content (%)			N/C Weight Ratio	Relative Atomic Amount of N Species		
	C	N	Fe		Pyridinic N	Pyrrolic N	Graphitic N
Fe_3_C@N/C-0	87.03	8.26	0.00	0.094	0.32	0.27	0.41
Fe_3_C@N/C-0.5	87.96	7.18	0.78	0.082	0.35	0.26	0.39
Fe_3_C@N/C-1	88.68	6.82	1.02	0.080	0.36	0.25	0.39
Fe_3_C@N/C-2	89.72	6.13	1.25	0.068	0.37	0.22	0.41

**Table 2:** The specific surface area and pore volume of Fe_3_C@N/C-x samples.

Sample	*S*_BET_	*V*_micro_
	(m^2^ g^−1^)	(cm^3^g^−1^)
Fe_3_C@N/C-0	2484.37	0.76
Fe_3_C@N/C-0.5	2121.87	0.71
Fe_3_C@N/C-1	1967.83	0.68
Fe_3_C@N/C-2	1687.45	0.58

**Table 3:** Summary of Fe_3_C-based electrocatalysts performance for oxygen-reduction reaction (ORR).

Catalyst	Electrolyte	Rotation Speed/rpm	Onset Potential/V vs. RHE	Diffusion Limiting Current (mA cm^−2^) vs. RHE	Ref.
PMF-800	0.1M KOH	1600	0.95	5.78	[29]
Fe_3_C/C-700	0.1M KOH	1600	0.89	4.21	[31]
Fe_3_C@NCNF-900	0.1M KOH	1600	0.93	4.51	[33]
Fe_3_C/NCNF	0.1M KOH	1600	1.012	4.81	[34]
Fe_3_C/b-NCNT	0.1M KOH	1600	0.96	6.25	[35]
Fe/C HN-700C-60M	0.1M KOH	1600	0.98	5.95	[45]
Fe@C-NG/NCNTs	0.1M KOH	1600	0.93	5.11	[46]
Fe_3_C@N/C-1	0.1M KOH	1600	0.94	5.78	This work
